# Variability of Steroid Prescription for COVID-19 Associated Pneumonia in Real-Life, Non-Trial Settings

**DOI:** 10.2478/jccm-2022-0025

**Published:** 2022-11-12

**Authors:** Rashid Nadeem, Islam Bon, Doaa Algohary, Mohd Kafeel Khan, Nilesh Gundawar, Mohammed Abdullah, Ekta Sharma, Moatz Galal Elzeiny, Mayada Mahmoud, Ashraf Elhoufi, Yusra Omar Alshaikh Sayed Ahmed, Gloria Gheno, Maged Talaat Salama Khalil, Tamseela Hussain

**Affiliations:** 1 Dubai Hospital, Dubai, UAE, United Arab Emirates; 2 Ronin Institute for Independent Scholarship, Montclair, New Jersey, USA

**Keywords:** COVID-19, infection, steroids, ARDS

## Abstract

The RECOVERY study documented lower 28-day mortality with the use of dexamethasone in hospitalized patients on invasive mechanical ventilation or oxygen with COVID-19 Pneumonia. We aimed to examine the practice patterns of steroids use, and their impact on mortality and length of stay in ICU. We retrospectively examined records of all patients with confirmed Covid 19 pneumonia admitted to the ICU of Dubai hospital from January 1st, 2020 – June 30th, 2020. We assigned patients to four groups (No steroids, low dose, medium dose, and high dose steroids). The primary clinical variable of interest was doses of steroids. Secondary outcomes were 28-day mortality and length of stay in ICU”. We found variability in doses of steroid treatment. The most frequently used dose was the high dose. Patients who survived were on significantly higher doses of steroids and had significantly longer stays in ICU. The prescription of steroids in Covid-19 ARDS is variable. The dose of steroids impacts mortality rate and length of stay in ICU, although patients treated with high dose steroids seem to stay more days in ICU.

## Introduction

Coronavirus disease 2019 (COVID-19) has caused approximately two million deaths worldwide as of June 30, 2021, including severe pneumonia cases associated with acute respiratory distress syndrome (ARDS) and respiratory failure requiring mechanical ventilation [1]. In moderate-to-severe cases, signs of organ dysfunction, such as ARDS, acute kidney injury, pulmonary edema, myocarditis, septic shock, and death can occur [2]. The RECOVERY study demonstrated that the use of dexamethasone decreased 28-day mortality in hospitalized patients on invasive mechanical ventilation or oxygen with Covid-19 pneumonia [3]. In contrast, the CAPE Covid-19 trial study did not show the beneficial impact of using low-dose hydrocortisone on the 21-day mortality rate in critically ill patients with Covid-19 [4]. Similarly, in the REMAP CAP study, low-dose hydrocortisone, compared to placebo, did not significantly reduce treatment failure at day 21 in critically ill COVID-19 patients with ARDS [5]. The METCOVID trial showed no mortality benefit with a short course of methylprednisolone in hospitalized COVID-19 patients [6].

During the Pandemic, clinicians practice patterns were influenced by variability in the reported results with different formulations and doses of steroids. In this study, we aimed to examine the impact of using a low, medium, or high dose of steroids on the function of 28-day survival and length of stay in the intensive care unit (LOSICU).

## Materials and Methods

This was a retrospective observational study of patients with confirmed COVID-19 admitted to the Dubai Hospital ICU. We excluded patients <18 years of age.

The primary variable of interest was steroid dosage. We recorded daily doses of steroids (hydrocortisone, methylprednisolone, or dexamethasone) for each patient throughout their ICU stay. Patients were categorized into four groups based on steroid dosage: no steroids, low dose, medium dose, and high dose. The definitions of dose categories are as follows: low dose (hydrocortisone [H], <200 mg/day; methylprednisolone [MP], <40 mg/ day; or dexamethasone [D], <12 mg/day), medium dose (H [200–400 mg/day], MP [40–80 mg/ day], or D [12–20 mg/day]), and high dose (H >400 mg/day, MP >80 mg/day, or D >20 mg/day). The primary clinical variables were dosages of steroid use. The clinical outcomes were 28-day survival from admission to ICU and LOSICU. Confounding factors affecting clinical outcomes were also recorded. The demographics recorded were as follows: age; sex; body mass index (BMI); nationality; clinical parameters; positive swab sample test result for viral DNA; the number of swabs; days to negative test result; the number of days of symptoms; and presence of symptoms, such as cough, fever, dyspnea, and gastric complaints. Data on comorbidities including diabetes, hypertension, coronary artery disease, renal failure, and outpatient dialysis were recorded. Inpatient clinical data on admission including fever, tachycardia, blood pressure, hypoxia, oxygen (L/ min), mechanical ventilation, use of pressers, and inpatient dialysis were also recorded. Laboratory parameters included inflammation markers (CRP, ferritin, and procalcitonin levels), hematologic indices (WBC and platelet counts), chemical tests (electrolyte levels), and culture studies for bacteremia since secondary bacterial infection impacts clinical outcomes. Bacterial infections included documented culture results of sputum, blood, pleural or peritoneal fluid, or pneumonia panel. Data on therapeutic agents, including chloroquine and antivirals, were also recorded since they could be significant confounding factors. We calculated Acute Physiology and Chronic Health Evaluation (APACHE-2) and it is designed for ICU mortality prediction (7). Age, gender, BMI, history of outpatient dialysis, presence of hypotension on admission to ICU are few variables used to balance the dataset using the propensity score.

The characteristics of all the patients included in this study and four categories of steroids doses are shown in Table 1 for categorical variables (except for genre, 0 represents the absence while 1 the presence of the analyzed factor) and in Table 2 for continuous variables (Q1 represents the first quartile, Q3 the third quartile).

**Table 1:** Sample characteristics (categorical variables)

**Categorical variables**	**Total Sample N 235**		**No steroids N 23**		**Low dose N 42**		**Medium dose N 23**		**High dose N 151**	
1= variable present	0	1	0	1	0	1	0	1	0	1
**Demographic recorded**										
Gender (F=0, M=1) (%)	13	87	3.8	17	2.9	7.6	1.3	7.1	4.6	55.5
Presence of symptoms										
cough	20	80	6.7	14.3	2.1	8.4	2.5	5.9	8.8	51.3
fever	9	91	2.5	18.5	1.3	9.2	0.4	8	5	55
dyspnea	20	80	7.6	13.4	1.3	9.2	1.7	6.7	9.7	50.4
Gastric complaints	88	12	18.5	2.5	8	2.5	7.1	1.3	54.6	5.5
**Comorbidities**										
diabetes	57	43	10.1	10.9	7.1	3.4	5.5	3	34.5	25.6
hypertension	75	25	15.5	5.5	6.3	4.2	5	3.4	48.3	11.8
coronary disease	93	7	18.5	2.5	8.8	1.7	8	0.4	58	2.1
renal failure	88	12	18.1	2.9	9.7	0.8	6.7	1.7	53.4	6.7
dialysis	93	7	19.7	1.2	9.7	0.8	7.6	0.8	56.3	3.8
**Inpatient clinical data on admission**										
Immunosuppressed	96	4	20.6	0.4	10.1	0.4	7.1	1.3	58.4	1.7
Fever	14	86	4.2	16.8	1.7	8.8	1.3	7.1	6.7	53.4
Tachycardia (pulse>100)	21	79	5.5	15.5	2.1	8.4	2.5	5.9	11.3	48.7
Hypotension on admission (MAP<60 mm of Hg)	50	50	10.9	10.1	6.7	3.8	4.2	4.2	28.2	31.9
Hypoxia on admission	13	87	5.9	15.1	0.8	9.7	0.8	7.6	5.9	54.2
Mechanical Ventilation	15	85	7.1	13.9	2.5	8	0.4	8	4.6	55.5
Vasopressors	21	79	10.1	10.9	3.4	7.1	0.8	7.6	6.7	53.4
Dialysis on admission	70	30	16.4	4.6	8.4	2.1	5	3.4	39.9	20.2
**Clinical parameters**										
Lymphopenia (≤ 1100 cells/μL)	44	56	12.2	8.8	7.1	3.4	2.9	5.5	21.8	38.2
Bacterial infection	49	51	16	5	9.2	1.3	3.4	5	20.2	39.9
Bacteremia	60	40	8.1	2.9	9.7	0.8	5.5	2.9	26.9	33.2
Positive cultures	45	55	15.1	5.9	8.8	1.7	2.5	5.9	18.1	42
Arrhythmia	15	22	15.5	5.5	8.8	1.7	6.7	1.7	47.1	13
**Associated treatment**										
Anticoagulants	4	96	2.9	18.1	0	10.5	0	8.4	0.8	59.2
Gastro-intestinal prophylaxis (Proton pump inhibitors)	4	96	2.1	18.9	0.8	9.7	0	8.4	1.3	58.8
**Therapeutic agent**										
Lopinavir/Ritonavir	6	94	3.4	17.6	1.3	9.2	0.4	8	1.3	58.8
Chloroquine	12	88	4.2	16.8	1.7	8.8	1.3	7.1	5	55
Lopinavir/Ritonavir	64	36	16.8	4.2	6.3	4.2	4.6	3.8	36.1	23.9
Favipiravir	20	80	10.5	10.5	2.5	8	1.3	7.1	5.9	54.2
Tociluzimab	84	16	20.2	0.8	9.7	0.8	6.7	1.7	47.5	12.6
Plasmatherapy	83	17	21	0	9.7	0.8	6.7	1.7	45.8	14.3
Tracheostomy	87	17	20.2	0.8	10.5	0	7.6	0.8	48.7	11.3
ECMO	95	5	21	0	10.5	0	7.6	0.8	55.5	4.6
Surgeries	96	4	20.6	0.4	10.5	0	8	0.4	56.7	3.4
Sedatives	11	89	6.7	14.3	1.7	8.8	0.4	8	2.5	57.6
Narcotics	24	76	9.7	11.3	1.7	8.8	2.1	6.3	10.5	49.6
Neuromuscular blocking agents	15	85	8.8	12.2	2.5	8	0.8	7.6	2.9	57.1

**Table 2:** Sample Characteristics Continuous Variables

	**Total N=235**		**No steroids N=23**		**Low dose N=42**		**Medium dose N=23**		**High dose N=151**	
**Continuous variables**	Median	IQR	Median	IQR	Median	IQR	Median	IQR	Median	IQR
**Demographic recorded**										
Age(years)	49	42-57	47	39-60	50	45-58	50	47-52	49	42-56
BMI (Kg/)	27	23-30	26	24-32	26	21-31	24	21-31	28	24-32
Days turn test negative	5	0-16	2	0-10	0	0-9	8	3-23	7	0-18
**Inpatient clinical data on admission**										
Oxygen (ml)	10	0-15	4	0-10	15	4-15	15	8.5-15	15	0.05-10
**Laboratory parameters**										
Ferritin (ng/ml)	1230	483-1895	468	120-1520	620	214-1804	1204	532-750	1385	750-1986
D-Dimer (ng/ml)	1.11	0.52-3.80	0.61	0.29-3.02	1.17	0.58-2.2	3.9	1.01-8.99	1.11	0.58-3.43
Procalcitonin (ng/ml)	0.35	0.14-1.168	0.27	0.1-2.26	0.21	0.1-1.23	0.31	0.15-0.98	0.37	0.16-1.10
CRP (mg/L)	129.5	75.2-215.88	104	64.8-216	133	73.4-161	98	59.6-156	137	84.5-225
Creatinine (mg/dl)	0.9	0.8-1.2	0.9	0.7-1.28	0.8	0.7-1.6	0.9	0.78-1.3	1	0.8-1.2
CPK (units/L)	196.5	69.25-626.50	120	17.8-439	164	52-410	310	141-609	218	86-689
ABG PH	7.37	7.25-7.43	7.35	0-7.43	7.3	7.19-7.38	7.40	7.35-7.44	7.37	7.28-7.43
PCo2 (Torr)	35.8	29.93-45.58	30	0-36.4	37.1	32.6-48.6	35.2	31.7-42.9	38	31.5-48.3
PO2 (Torr)	61.3	44.30-84.95	48.7	0-88.7	64	47-73	61.8	45.5-75	62	47.4-85.7
Lactate	1.7	1.2-2.5	1.35	0.23-2.28	2.4	1.4-6.3	1.9	1.37-3.47	1.7	1.2-2.3
Bicarbonate (mEq/L)	21.55	18.8-24	20	14.2-22.5	20.7	16.3-23.9	21.4	19.7-23.6	22.1	19.5-24.4
Magnesium (mg/dl)	2.03	1.84-2.24	2.01	1.72-2.16	1.94	1.7-2.24	2.09	1.87-2.39	2.04	1.90-2.26
Platelets (/microliter)	196.5	150.25-260.5	206	158-252	214	141-299	164	126-253	197	152-262
WBC (/microliter)	7.9	6.03-10.7	7.9	5.9-10.8	8.1	6.3-13	7.15	6.15-9.85	7.9	6-10.7
**Severity of illness**										
APACHE 2 score	16	12-20	15.5	2.25-24.5	16	13-22	18.5	13-24.2	16	12-19
**Variable of interest**										
Days on Mech. Vent.	10	4-20	4	0-8.75	2	1-9	15	7.75-23	14	8-23
LOSICU (days)	12	4-22.75	4	0-8.75	2	1-9	17	8.75-23.8	18	10-30.5
LOS in hospital (days)	18	8-32	10	6.25-18.8	5	2-12	28	13.8-49.5	22	12.5-35.5

### Statistical analysis

All analyses were performed with R-project (R Core Team, 2021, R: a language and environment for statistical computing. R Foundation for Statistical Computing, Vienna, Austria), We used the Cox proportional hazard model (Cox, 1972) to analyze survival time as it is a multivariate statistical model for censored time-to-event data. In this model, the dependent variable can be defined as the risk for death at that moment or as the probability of dying given that patients have survived up to a given point in time. We used Cox proportional model as it permits the introduction of many other factors (called covariates or regressors) such that it is possible to analyze the effectiveness of 2 or more different treatments considering additional factors, which can influence the risk of death.

The quantity, where is the parameter of the covariate X, is called hazard ratios (HR). A value of greater than zero, or equivalently a hazard ratio greater than one, indicates that as the value of the covariate X increases, the risk of death increases.

## Results

Our sample predominantly consist of young male (87%). More than 80% of patients presented with cough, fever and difficulty breathing. Less than half had a comorbid condition (Diabetes-43%, hypertension-25% and coronary artery disease-7%). More than 75% had fever, tachycardia, and hypoxemia on admission. On admission to ICU 87% required mechanical ventilation and 79% required vasopressors. More than half developed secondary bacterial infection. About 55% grew positive culture from sputum, urine, or blood (bacteremia in 40%). Prophylactic anticoagulation and gastrointestinal prophylaxis were used in 96%.

The patients with medium or high doses had a longer stay (days) on the ventilator (14 versus 4), in the ICU (18 vs 4), and in the hospital (22 vs 10). Higher doses were more frequently administered in survivors.

**Fig. 1:**
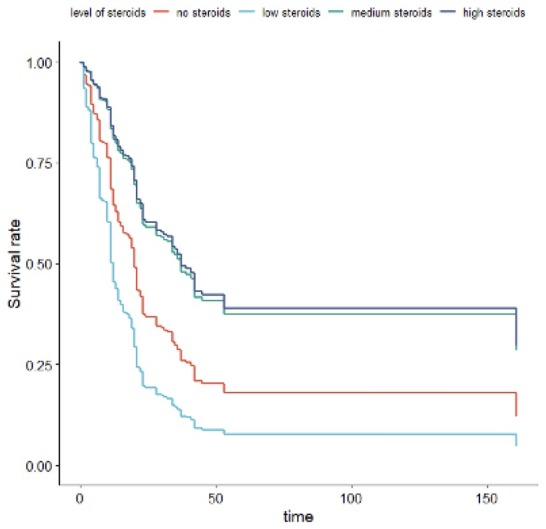
Length of stay in hospital (LOS)

**Fig. 2:**
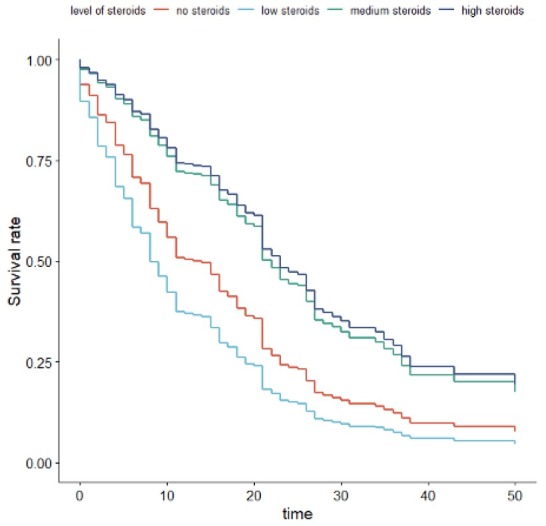
Days of stay in ICU

Bacteremia, treatment with chloroquine, lopinavir/ Ritonavir, are found to be predictors of mortality rate. Levels of Ferritin, and CRP were also predictors of mortality rate. Use of BIPAP and longer stay on Ventilators, tachycardia, use of Favipiravir, chloroquine, Lopinavir/Ritonavir, tracheostomy, and plasma therapy, were found to be predictors of mortality rate considering LOSICU. Survival was better for patients treated with high or medium dose of steroids (Figure 1-2).

If we consider the “Days in ICU,” the best therapy is the high dose and the second one is the medium dose: a high dose decreases the risk of death by 64% while a medium dose by 62%. The parameters of the medium dose and high dose are respectively -1.08405 and -1.21542 (|-1.08405|<|-1.21542|). If we consider the “LOS in hospital,” we obtain similar results. The parameters of the medium dose and high dose are respectively -0.96653 and -1.02833. Also, in this case, both are negative, and this means that the risk of death decreases. The decrease is bigger for the high dose than the medium dose i.e., a high dose decreases the risk of death by 70% while a medium dose by 66%.

In observational studies, the treatment allocation is not random, and to obtain unbiased results it is necessary to use sophisticated statistical methods such as propensity score.

## Discussion

We hypothesize that the reasons for variability in our study reflect how research translates into clinical practice. It is multifactorial and includes physicians’ bias (8), cost of treatment, side effects of steroids, fear of the steroid’s impact on the behavior of co-existing infections (9), and steroid dependency. Moreover, there may be other indications that could influence the duration of steroid use. In contrast, research trials restrict steroid use as per protocol; therefore, the doses are fixed, and treatment has a clearly defined course [3,6,10]. Improved survival in patients with high CRP levels and intensity of inflammatory cytokines may have influenced clinicians to increase the dose or duration of steroid use [11]. We found that the secondary bacterial infection impacts mortality, similar observations have been documented by Garcia et al [12]. Continuous renal replacement therapy (CRRT) was not a predictor of mortality in our study in contrast to the study by Ng et al. [13]. Published studies on steroid use did not include extensive data on confounding factors. Therefore, we believe our inclusion of a higher number of potential confounders, and statistical modeling to adjust the impact of these confounding factors provided more accurate results. The prospective design of studies such as the RECOVERY trial [3] allows the allocation of patients in both groups (steroids versus no steroids), however, confounders, like new bacterial infections appear later in the course of the disease which could have a significant impact on outcomes. Adjusting for such dynamic factors could change outcome calculations. Similarly, most of these patients do not have renal impairment requiring CRRT at presentation to hospital, therefore prospective trials may have calculated outcomes without adjustment of this significant factor affecting survival. Therefore, retrospective design with appropriate modeling may provide a better assessment of the effect of confounding variables upon outcome measures. Therefore, we believe our results are specific and attributable to steroid usage.

Patients treated with higher doses of steroids stayed longer on mechanical ventilation, in the ICU and in the hospital. This could result from multiple factors. Patients treated with lower doses had higher mortality hence the shorter duration of mechanical ventilation (MV) and stay in ICU. Patients treated with higher doses are more likely to develop myopathy [14], gastrointestinal bleeding [15], electrolytes disturbances, poor wound healing [16] and psychosis [17] that may increase morbidity and prolong need for MV, ICU and hospital stay.

Bacteremia was predictor of mortality in our sample. We had 84 incidences of bacteremia including 11 catheter related bacteremia. half of them were gram negative bacteremia with Pseudomonas Aeroginosa being the most common while 48% were gram positives with predominantly Staphylococcus Aureus. We published the details of these recently [18]. Mylotte et. al. [19] also found comparable results. Measures of severity of illness (Ferritin and CRP) were significant predictors of mortality in our sample just as others found similar results [20, 21]. Tracheostomy was associated with better survival in our study. Comparable results were reported by Ahmed et. al. [22]. Antiviral (Favipiravir, Lopinavir/Ritonavir) were also associated with better survival in our study. Bayrak et. al [23] found Favipiravir is associated with good outcome while a large study showed no mortality benefit from Lopinavir/Ritonavir [24].

Another interesting observation in our study was ‘delayed viral clearance’ in survivors who received steroid treatment. A high proportion of those patients who survived were on steroid treatment, suggesting steroids may delay viral clearance owing to the process of viral clearance possibly dependent upon the strength of inflammatory response. However, we would like to caution the reader that we did not measure viral loads therefore we cannot validate that steroid specifically delay viral clearance. Delayed viral clearance has been documented in patients with SARS-CoV-1 and MERS-CoV infections in patients not requiring oxygen supplementation [10]. Such delays in viral clearance may affect outcomes.

We identify the following limitations: it is a single-center, retrospective study with a small sample size in which multiple forms of steroids were used. The population was predominantly young males. Therefore, the findings of this study may not be generalizable to other communities. There was no standard protocol determining dose and duration, although we will argue that this was our primary aim, i.e., to measure real, non-trial patterns of steroid use without the strict control of the use of one drug and fixed dose. To the best of our knowledge, variability in steroid dose patterns has not been previously documented in COVID-19 patients. Existing data include steroid treatment practice on admission in a research clinical trial setting. Our clinical observations suggest that steroid doses are frequently changed due to dynamic clinical profile, i.e., development of shock, secondary infection, and complications such as gastrointestinal bleeding.

## Conclusions

The prescription of steroids in COVID-19 patients with ARDS is variable. A high or medium dose of steroids predicts survival and length of stay in ICU. Future studies should explore these observations and define the optimal dosing and duration of steroids; to reduce variability in clinical practice and maximize clinical benefits.

## Data Availability Statement

The data that support the findings of this study are available on request from the corresponding author. The data are not publicly available due to privacy or ethical restrictions.

## References

[1] Mallah S.I., Ghorab O.K., Al-Salmi S. (2021). COVID-19: breaking down a global health crisis. Annals of clinical microbiology and antimicrobials.

[2] Huang C, Yeming W, Xingwang L (2020). “Clinical features of patients infected with 2019 novel coronavirus in Wuhan, China. ” The Lancet.

[3] Horby P., Lim W.S., Landray M.J. (2021). Dexamethasone in hospitalized patients with Covid-19. The New England journal of medicine.

[4] Dequin PF., Heming N., Meziani F. (2020). Effect of hydrocortisone on 21-day mortality or respiratory support among critically ill patients with COVID-19: a randomized clinical trial. Jama.

[5] Angus DC, Derde L, Al-Beidh F (2020). Effect of hydrocortisone on mortality and organ support in patients with severe COVID-19: the REMAP-CAP COVID-19 corticosteroid domain randomized clinical trial. Jama.

[6] Jeronimo CM, Farias ME, Val FF (2021). Methylprednisolone as adjunctive therapy for patients hospitalized with coronavirus disease 2019: a randomized, double-blind, phase IIb, placebo-controlled trial. Clinical Infectious Diseases.

[7] Knaus WA, Draper EA, Wagner DP, Zimmerman JE (1985). APACHE II: a severity of disease classification system. Critical care medicine.

[8] Chapman EN, Kaatz A, Carnes M (2013). Physicians and implicit bias: how doctors may unwittingly perpetuate health care disparities. Journal of general internal medicine.

[9] Obata R, Maeda T, Rizk D, Kuno T (2021). Increased secondary infection in COVID-19 patients treated with steroids in New York City. Jpn J Infect Dis.

[10] Li H, Chen C, Hu F (2020). Impact of corticosteroid therapy on outcomes of persons with SARS-CoV-2, SARS-CoV, or MERS-CoV infection: a systematic review and meta-analysis. Leukemia.

[11] Xu K, Chen Y, Yuan J (2020). Factors associated with prolonged viral RNA shedding in patients with COVID-19. Clin Infect Dis.

[12] Garcia-Vidal C, Sanjuan G, Moreno-García E (2021). Incidence of co-infections and superinfections in hospitalized patients with COVID-19: a retrospective cohort study. Clinical Microbiology and Infection.

[13] Ng JH, Hirsch JS, Wanchoo R (2020). Outcomes of patients with end-stage kidney disease hospitalized with COVID-19. Kidney Int.

[14] Bowyer SL, LaMothe MP, Hollister JR (1985). Steroid myopathy: incidence and detection in a population with asthma. Journal of allergy and clinical immunology.

[15] Narum S, Westergren T, Klemp M (2014). Corticosteroids and risk of gastrointestinal bleeding: a systematic review and meta-analysis. BMJ open.

[16] Goforth P, Gudas CJ (1980). Effects of steroids on wound healing: a review of the literature. The Journal of foot surgery.

[17] Ravindran NP, Halder A, Harshe D, Harshe S, Harshe G (2022). A qualitative analysis of literature reporting and linking psychosis to COVID-19 infection. Asian journal of psychiatry.

[18] Nadeem R, Aijazi I, Elhoufi A (2021). Clinical Profile of Mortality and Treatment Profile of Survival in Patients with COVID-19 Pneumonia Admitted to Dubai Hospital. Dubai Medical Journal.

[19] Mylotte JM, Tayara A (2000). Staphylococcus aureus bacteremia: predictors of 30-day mortality in a large cohort. Clinical Infectious Diseases.

[20] Lino K, Guimarães GM, Alves LS (2021). Serum ferritin at admission in hospitalized COVID-19 patients as a predictor of mortality. Brazilian Journal of Infectious Diseases.

[21] Ali A, Noman M, Guo Y (2021). Myoglobin and C-reactive protein are efficient and reliable early predictors of COVID-19 associated mortality. Scientific reports.

[22] Ahmed Y, Cao A, Thal A (2021). Tracheotomy outcomes in 64 ventilated COVID‐19 patients at a high‐volume center in Bronx, NY. The Laryngoscope.

[23] Bayrak V, Durukan NŞ, Aydemir FD (2021). Risk factors associated with mortality in ıntensive care COVID-19 patients: the importance of chest CT score and intubation timing as risk factors. Turkish journal of medical sciences.

[24] Lora-Tamayo J, Maestro G, Lalueza A (2021). Early Lopinavir/ ritonavir does not reduce mortality in COVID-19 patients: Results of a large multicenter study. Journal of Infection.

